# The Use of Acute Immunosuppressive Therapy to Improve Antibiotic Efficacy against Intracellular Staphylococcus aureus

**DOI:** 10.1128/spectrum.00858-22

**Published:** 2022-05-16

**Authors:** Jenna E. Beam, Sophie Maiocchi, Ana Cartaya, Sarah E. Rowe, Edward S. M. Bahnson, Brian P. Conlon

**Affiliations:** a Department of Microbiology and Immunology, University of North Carolina at Chapel Hill, Chapel Hill, North Carolina, USA; b Department of Cell Biology & Physiology, University of North Carolina at Chapel Hill, Chapel Hill, North Carolina, USA; c Curriculum in Toxicology & Environmental Medicine, University of North Carolina at Chapel Hill, Chapel Hill, North Carolina, USA; d Center for Nanotechnology in Drug Delivery, University of North Carolina at Chapel Hill, Chapel Hill, North Carolina, USA; e McAllister Heart Institute, University of North Carolina at Chapel Hill, Chapel Hill, North Carolina, USA; f Department of Pharmacology, University of North Carolina at Chapel Hill, Chapel Hill, North Carolina, USA; g Marsico Lung Institute, University of North Carolina at Chapel Hill, Chapel Hill, North Carolina, USA; Emory University School of Medicine

**Keywords:** antibiotics, persister cells, reactive oxygen species, *Staphylococcus aureus*, immunomodulation

## Abstract

Interactions between Staphylococcus aureus and the host immune system can have significant impacts on antibiotic efficacy, suggesting that targeting and modulating the immune response to S. aureus infection may improve antibiotic efficacy and improve infection outcome. As we’ve previously shown, high levels of reactive oxygen species (ROS), associated with an M1-like proinflammatory macrophage response, potently induce antibiotic tolerance in S. aureus. Although the proinflammatory immune response is critical for initial control of pathogen burden, recent studies demonstrate that modulation of the macrophage response to an anti-inflammatory, or M2-like, response facilitates resolution of established S. aureus skin and soft tissue infections, arthritis, and bacteremia. Here, we evaluated the impact of host-directed immunosuppressive chemotherapeutics and anti-inflammatory agents on antibiotic efficacy against S. aureus.

**IMPORTANCE** Staphylococcus aureus is the leading cause of hospital-acquired infections in the United States with high rates of antibiotic treatment failure. Macrophages represent an important intracellular niche in experimental models of S. aureus bacteremia. Although a proinflammatory macrophage response is critical for controlling infection, previous studies have identified an antagonistic relationship between antibiotic treatment and the proinflammatory macrophage response. Reactive oxygen species, produced by macrophages during respiratory burst, coerce S. aureus into an antibiotic tolerant state, leading to poor treatment outcome. Here, we aimed to determine the potential of host-directed immunomodulators that reduce the production of reactive oxygen species to improve antibiotic efficacy against intracellular S. aureus.

## OBSERVATION

The formation of antibiotic tolerant persister cells has historically been studied under *in vitro* conditions, which fail to recapitulate the complex host environment. In experimental models of Staphylococcus aureus bacteremia, bacteria are engulfed by macrophages within minutes of entering the bloodstream, leading to a potent proinflammatory immune response associated with increased NF-κB activity (1, 2). Upon phagocytosis, S. aureus is exposed to an array of bactericidal assaults, including production of reactive oxygen species (ROS) during oxidative burst (3). Multiple species are generated during oxidative burst, including superoxide by the NADPH oxidase (NOX2) complex, nitric oxide by inducible nitric oxide synthase (iNOS), hydrogen peroxide, and peroxynitrite, produced by the reaction of superoxide and nitric oxide (4). Although these strategies are designed to kill S. aureus, macrophages often fail to eradicate the infection. Infected macrophages may then function as “Trojan horses,” shielding S. aureus from antibiotic and immune-mediated clearance, facilitating S. aureus dissemination to other tissues, often resulting in secondary infections (1, 5).

We and others have previously shown that macrophage-derived ROS, specifically peroxynitrite, induces metabolic indolence and consequent antibiotic tolerance in S. aureus (4, 6, 7). Our group found that peroxynitrite damages S. aureus aconitase, preventing TCA cycle flux, leading to low ATP levels and subsequent antibiotic tolerance (4). High levels of peroxynitrite, and ROS in general, are associated with an M1-like proinflammatory macrophage response (7, 8). Although the proinflammatory macrophage response is critical for initial control of pathogen burden, the bacteria that survive may be coerced into an antibiotic tolerant state driven by ROS-mediated damage. Thus, in this case, modulation of the immune response toward an anti-inflammatory “M2-like” response, where ROS production is decreased, could improve antibiotic efficacy.

Modulation of the macrophage response to an M2-like response was shown to facilitate resolution of S. aureus skin and soft tissue infections (SSTIs) and bacteremia via upregulation of peroxisome proliferator-activated receptor gamma (PPARγ) (9, 10). Corticosteroids, such as dexamethasone, are immunosuppressive agents that have been shown to improve antibiotic efficacy against bacterial infections. In a S. aureus arthritis model, co-administration of dexamethasone and antibiotics significantly decreased disease severity and resulted in rapid resolution of infection compared to antibiotic treatment alone (11). Additionally, activation of nuclear erythroid–related factor 2 (Nrf2) signaling has been shown to improve bacterial clearance by alveolar macrophages in patients with chronic obstructive pulmonary disorder (COPD) (12). Nrf2 is a transcription factor that drives expression of antioxidants and anti-inflammatory cytokines during the resolution phase of the immune response (13, 14). Increased Nrf2 signaling has been shown to attenuate the NF-κB inflammatory response and decrease iNOS expression and ROS production in macrophages (13–15).

Here, we evaluate the impact of host-directed immunosuppressive chemotherapeutics and anti-inflammatory agents on antibiotic efficacy against intracellular S. aureus.

### Results: treatment of macrophages with dexamethasone and rosiglitazone improves antibiotic efficacy against S. aureus.

**FIG 1:**
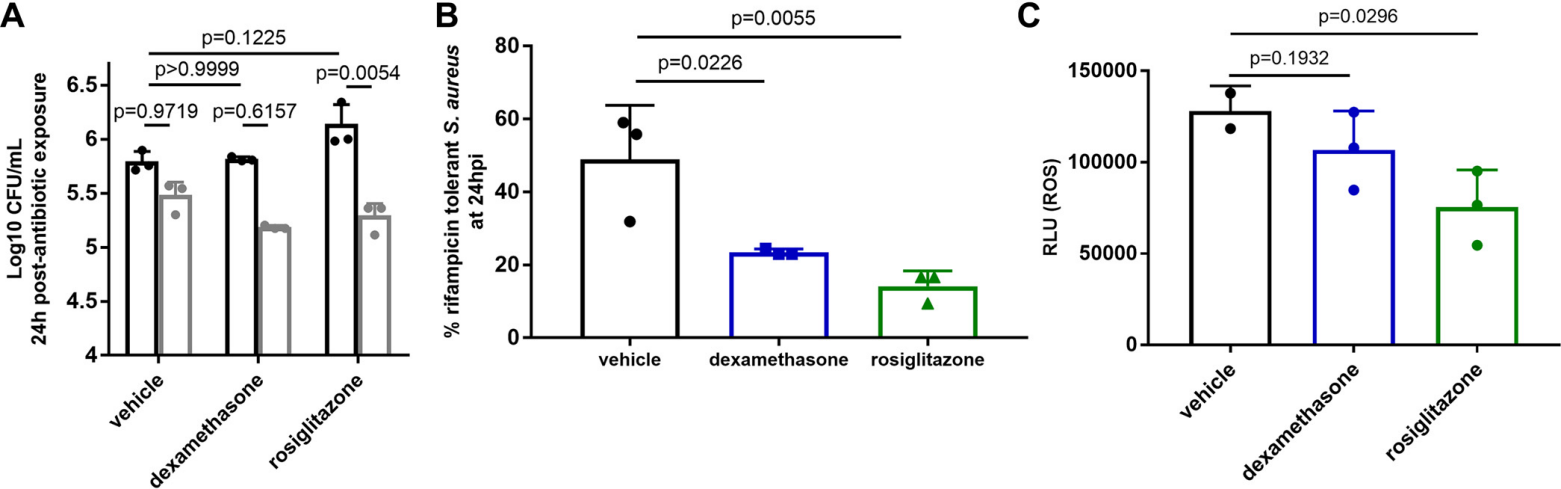
Treatment of macrophages with the immunomodulators dexamethasone and rosiglitazone improves antibiotic efficacy against S. aureus. (A) CFU of S. aureus recovered from BMDMs treated with dexamethasone or rosiglitazone, followed by treatment with rifampicin. Black bars, no antibiotic; gray bars, rifampicin. (B) % survival extrapolated from (A). (C) Relative light units (RLU) of L012 (proxy for ROS) in macrophages treated as in (A). Data are representative of *n* = 3 biological samples. Error bars represent standard deviation. Statistical significance was determined using one-way ANOVA with Sidak’s multiple comparison test. There is no significant difference in S. aureus killing in the absence of rifampicin (compare black bars in A).

Corticosteroids are largely immunosuppressive drugs, although their effects on monocytes and macrophages are complex (16). To determine the impact of corticosteroid treatment on antibiotic efficacy against S. aureus, we treated bone marrow-derived macrophages (BMDMs) with the corticosteroid dexamethasone prior to infection with S. aureus and treatment with rifampicin. While treatment of macrophages with dexamethasone alone did not affect bacterial load, in combination with rifampicin, it significantly improved antibiotic efficacy against S. aureus after 24 h, which correlated with decreased ROS production (Fig. 1A to C).

Rosiglitazone was previously shown to improve immune-mediated clearance of an S. aureus SSTI via agonism of PPARγ, a lipid metabolism regulator that leads to decreased iNOS expression and overall ROS levels in macrophages, which, according to our prior findings, should increase antibiotic efficacy (9). However, the impact of rosiglitazone on antibiotic susceptibility of S. aureus was not determined. Thus, to determine if rosiglitazone treatment increases antibiotic susceptibility of S. aureus, we treated BMDMs with rosiglitazone prior to infection with S. aureus and treatment with rifampicin. Treatment of macrophages with rosiglitazone alone did not affect bacterial load. However, in combination with rifampicin, it significantly improved antibiotic efficacy against S. aureus after 24 h, which correlated with decreased ROS production (Fig. 1A to C). Neither dexamethasone nor rosiglitazone had any direct affect on the minimal inhibitory concentration of rifampicin (Table 1).

**TABLE 1 tab1:** MIC of rifampicin in S. aureus strain LAC^*a*^

Drug	MIC (μg/mL)
Rifampicin	0.004
Rifampici*n* + 10μM rosiglitazone	0.004
Rifampici*n* + 10μM sulforaphane	0.004
Rifampici*n* + 400nM CDDO-Me-NPs	0.004
Rifampici*n* + 100nM dexamethasone	0.004

a MIC assays for rifampicin were conducted using a microdilutionmethod. Approximately 5 × 10^5^ LAC cells in Mueller-Hinton broth were incubated with various concentrations of rifampicin in a 96-wellmicrotiter plate. Where indicated, wells were supplemented with 100mM rosiglitazone, 10mMsulforaphane, 400 nM CDDO-Me-NPs, 100 nM dexamethasone. The plate was covered with a breath easy strip and incubated for 24h at 37°C. The MIC of rifampicin was 0.004mg/mL, and was unchanged by supplementation with rosiglitazone, sulforaphane, CDDO-Me-NPs or dexamethasone. The experiment was performed in biological triplicates.

### Nrf2 signaling activation increases antibiotic susceptibility of S. aureus.

**FIG 2:**
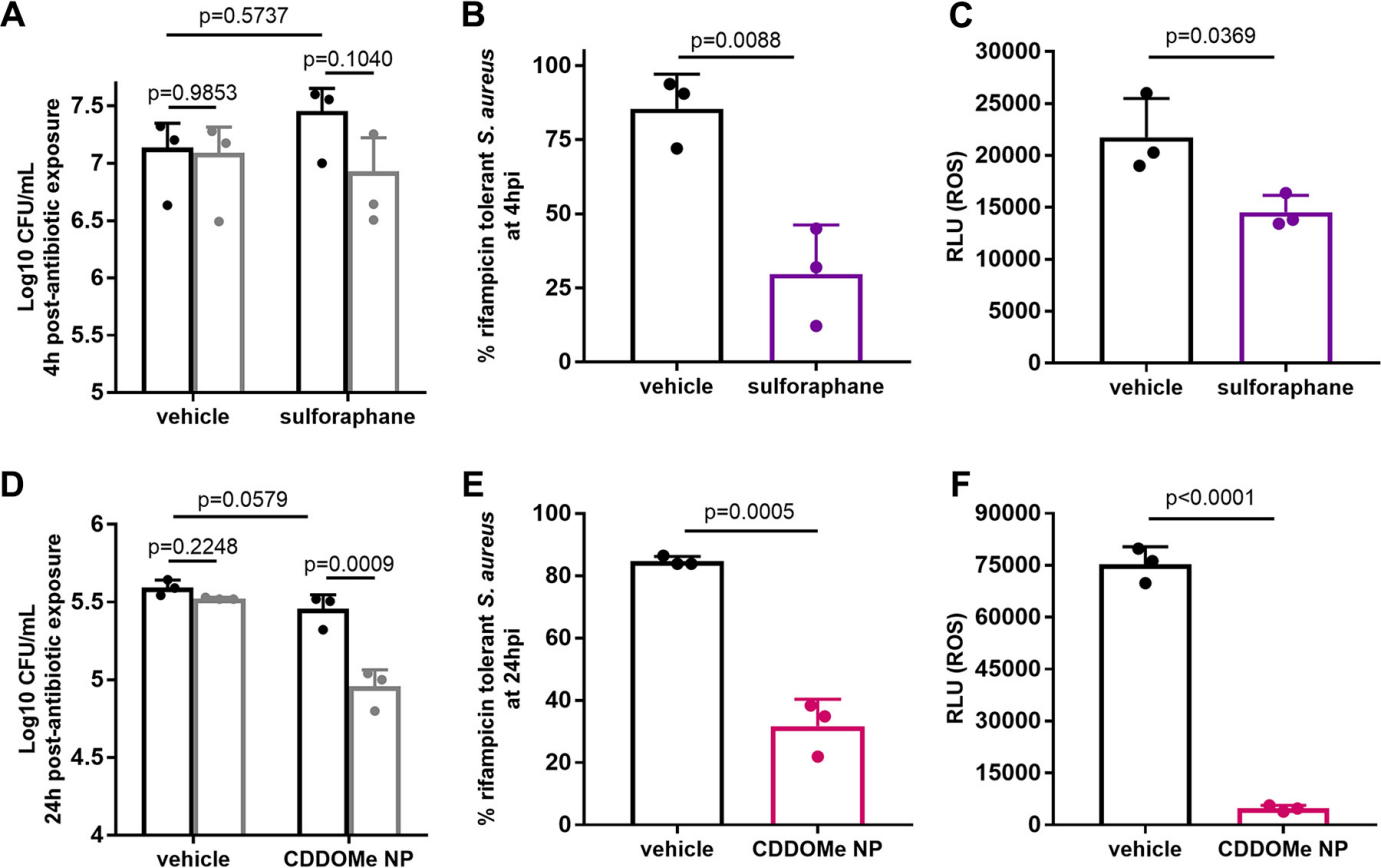
Treatment of macrophages with Nrf2 signaling activators improves antibiotic efficacy against S. aureus. (A, D) CFU of S. aureus recovered from BMDMs treated with sulforaphane (A) or CDDOMe ARAPas (D), followed by treatment with rifampicin. (B, E) % survival extrapolated from (A, D respectively). (C, F) Relative light units (RLU) of L012 (proxy for ROS) in macrophages treated as in (A, D respectively). Data are representative of *n* = 3 biological samples. Error bars represent standard deviation. Statistical significance was determined using Student’s *unpaired t test*. There is no significant difference in S. aureus killing in the absence of rifampicin (compare black bars in A, D).

As Nrf2 signaling leads to decreased ROS levels, we hypothesized that activation of Nrf2 signaling would increase antibiotic susceptibility of S. aureus in macrophages. To test this, we employed two Nrf2 activators: sulforaphane (17) and CDDO-methyl (CDDOMe; also called bardoxolone methyl) encapsulated in Antioxidant Response Activating nanoParticles (ARAPas) (15) CDDOMe ARAPas were recently shown to target to macrophages and block inflammatory signaling (15) To determine if Nrf2 signaling increases antibiotic susceptibility of S. aureus, BMDMs were treated with either sulforaphane (Fig. 2A to C) or CDDOMe ARAPas (Fig. 2D to F) prior to infection with S. aureus and treatment with rifampicin. Treatment with either sulforaphane or CDDOMe ARAPas did not affect bacterial survival 24 h postexposure in the absence of antibiotic (Fig. 2A and D). However, in combination with rifampicin, both significantly increased the antibiotic susceptibility of S. aureus (Fig. 2B and E), which correlated with decreased ROS production (Fig. 2C and F). Neither suphorophane nor CDDO-Me-NPs had any direct affect on the minimal inhibitory concentration of rifampicin (Table 1). Together, these data demonstrate that activation of the Nrf2 signaling pathway improves antibiotic efficacy.

### Discussion.

Host-pathogen interactions have been shown to have significant impacts on antibiotic treatment outcome. Although the proinflammatory immune response is critical for control of pathogen burden, we and others have demonstrated the role of a proinflammatory immune response in the induction of antibiotic tolerance (4, 6, 7, 18–20). Herein, we suggest that acute immunosuppressive therapy and inhibition of inflammation will improve antibiotic efficacy against intracellular S. aureus.

Both corticosteroids and PPARγ agonists have been shown to impact the phenotype of macrophages during infection (9, 11). Even though both dexamethasone and rosiglitazone improved antibiotic efficacy, only the PPARγ agonist significantly reduced ROS. This suggests that dexamethasone could act through a ROS-independent anti-inflammatory mechanism. The PPARγ agonist rosiglitazone stimulates an M2-like macrophage response, characterized by downregulation of iNOS, while dexamethasone treatment resulted in decreased immune cell migration to the infection site and reduced serum nitrate levels (9, 11). Additionally, activation of Nrf2 signaling has been shown to decrease inflammation and skew macrophages toward an M2-like state (17). In a study comparing various Nrf2 activators, sulforaphane was shown to have the greatest effect on decreasing inflammation and improving macrophage bacterial clearance (17). Furthermore, dimethyl fumarate, another Nrf2 signaling activator, was shown to promote clearance of uropathogenic E. coli in a mouse model of urinary tract infection (UTI) (21). UTIs are often recalcitrant to antibiotic treatment, underpinning the utility of host-directed therapeutics against bacterial infections. While neither of these studies analyzed the effects of Nrf2 activation on antibiotic efficacy, they lend support to our hypothesis that acute immunosuppressive therapy can positively impact treatment outcome.

Both Nrf2 activators tested decreased ROS and increased antibiotic efficacy. However, the CDDO-Me ARAPas decreased bacterial load to a greater extent. Nrf2 activators do not present a typical dose response curve, in fact most of them show a hormetic curve where high doses result in inhibition of the pathway (22). Because we did not perform a dose equivalence between sulforaphane and ARAPas, we don’t know where in their respective hormetic curves our doses fall, which could explain differences in the results obtained with sulforaphane versus CDDO-Me ARAPas.

A study of S. aureus-induced experimental endophthalmitis tested the effect of combinatorial antibiotic and dexamethasone treatment on bacterial clearance, tissue damage, and retinal function (23). Compared with dexamethasone or antibiotics alone, combination therapy decreased inflammation, which in turn preserved retinal function and decreased tissue damage. Compared with antibiotics alone, combination treatment also improved bacterial clearance (23). Interestingly, however, a study analyzing the effects of corticosteroids on the pharmacological activity of different antibiotics against *in vitro* biofilms found decreased activity against S. aureus by MIC assay of chloramphenicol, oxacillin, ceftriaxone, but not gentamicin or meropenem (24). A different study found that dexamethasone did not impact the efficacy of moxifloxacin against S. aureus in a model of aortic valve endocarditis (25), suggesting that the effects of dexamethasone on antibiotic activity may be specific to certain classes of antibiotics and/or certain infection types. While these studies together identify some of the potential obstacles of combination therapy in patients, they simultaneously highlight the importance of studying antibiotic efficacy in the context of host infection, and provide evidence that combination immunosuppressive therapy may improve patient outcomes.

Further studies evaluating the therapeutic efficacy of these compounds are still needed, including determination of any off-target effects and subsequent outcome on antibiotic efficacy. To that end, evaluation of the efficacy of these compounds in combination with other antibiotics remains to be determined. Rifampicin was chosen due to its ability to readily penetrate the intracellular environment; however, vancomycin, sometimes in combination with rifampicin, is the frontline treatment for S. aureus infection (26). Though vancomycin doesn’t penetrate into host cells (27), how immunomodulation impacts the killing of extracellular S. aureus is also of interest. Additionally, investigation of the effects of these compounds on other macrophage phenotypes, as well as on other immune cells, remains to be determined as they pertain to antibiotic treatment. However, altogether, the results presented here identify the potential of acute immunomodulation to improve antibiotic efficacy against intracellular S. aureus.

### Materials and methods: bacterial strains and growth conditions.

S. aureus strain LAC (28) was cultured in Mueller-Hinton broth (MHB) at 37°C and 225 rpm.

### BMDM isolation and infection.

Bone marrow from wildtype (WT) C57BL/6J mice (Jackson Laboratory) was isolated as described in (29). Bone marrow cells were differentiated for 7 days in Dulbecco’s Modified Eagle Medium (DMEM) + 10% FBS + l-glutamine + sodium pyruvate + sodium bicarbonate + 30% L929-conditioned media. After 7 days, cells were plated at 4 × 10^5^ cells/mL in minimum essential media (MEM) + 10% FBS + l-glutamine (complete MEM) and allowed to adhere overnight at 37°C, 5% CO_2_. BMDMs were treated with 10 μM sulforaphane (17), 400 nM CDDOMe ARAPas (15), 100 nM dexamethasone (30), or 10 μM rosiglitazone (9) overnight. BMDMs were incubated with S. aureus strain LAC at MOI 10 for 45 min at 37°C, 5% CO_2_ to allow for internalization. Media was removed, cells were washed one time with PBS, and media was replaced with complete MEM + gentamicin 50 μg/mL and/or rifampicin 10 μg/mL. At indicated times, macrophages were lysed with 0.1% Triton X-100 to release the bacteria. PBS was added to each well, lysates were resuspended by pipetting, serially diluted in 1% NaCl and plated to enumerate surviving bacteria. Percent survival after rifampicin treatment was determined by comparing survivors after 24 h antibiotic treatment to survivors of the corresponding untreated time point. Averages and standard deviations of three biological replicates are shown (*n* = 3). Statistical significance was calculated using the Student’s *t* test (unpaired, two-tailed) or one-way ANOVA with Sidak’s multiple comparison test as described in the figure legends.

### ROS measurements.

The luminescent probe L-012 (Wako Chemical Corporation) was used to measure ROS. BMDMs were seeded at 4 × 10^4^ cells per well in white tissue-culture-treated 96-well plates. Macrophages were treated as described above. The cells were washed three times with PBS. L-012 was diluted to 150 μM in Hanks’ balanced salt solution (Gibco). Luminescence was read immediately using a Biotek Synergy H1 microplate reader. Data shown are representative of three independent assays of three biological replicates. Statistical significance was calculated using a one-way ANOVA with Sidak’s multiple comparison test or Student’s *unpaired t test* as indicated in figure legends.

### Minimal inhibitory concentration assays.

MIC assays for rifampicin were conducted using a microdilution method. Approximately 5 × 10^5^ LAC cells in Mueller-Hinton broth were incubated with various concentrations of rifampicin in a 96-wellmicrotiter plate. Where indicated, wells were supplemented with 100mM rosiglitazone, 10mMsulforaphane, 400 nM CDDO-Me-NPs, 100 nM dexamethasone. The plate was covered with a breath easy strip and incubated for 24h at 37°C. The MIC of rifampicin was 0.004mg/mL, and was unchanged by supplementation with rosiglitazone, sulforaphane, CDDO-Me-NPs or dexamethasone. The experiment was performed in biological triplicates.

### Statistical information.

Statistical method and sample size (*n*) are indicated in the methods for each experiment. Statistical analysis was performed using Excel (Microsoft) or Prism 8 (GraphPad) software.

### Data availability.

Additional data that support the findings of this study are available from the corresponding [Table tab1]author.
